# The Application of a Self-Made Integrated Three-in-One Microsensor and Commercially Available Wind Speed Sensor to the Cold Air Pipe of the Heating, Ventilation, and Air Conditioning in a Factory for Real-Time Wireless Measurement

**DOI:** 10.3390/s23094471

**Published:** 2023-05-04

**Authors:** Chi-Yuan Lee, Jiann-Shing Shieh, Jerry Chen, Xin-Wen Wang, Chen-Kai Liu, Chia-Hsin Wei

**Affiliations:** Department of Mechanical Engineering, Yuan Ze Fuel Cell Center, Yuan Ze University, Taoyuan 32003, Taiwan

**Keywords:** self-made integrated three-in-one microsensor, commercially available wind speed sensor, cold air pipe of heating, ventilation, and air conditioning, real-time wireless measurement

## Abstract

In this study, the integrated three-in-one (temperature, humidity, and wind speed) microsensor was made through the technology of the Micro-electro-mechanical Systems (MEMS) to measure three important physical quantities of the internal environment of the cold air pipe of the Heating, Ventilation and Air Conditioning (HVAC) in the factory, plan the installation positions of the integrated three-in-one microsensor and commercially available wind speed sensor required by the internal environment of the cold air pipe, and conduct the actual 310-h long term test and comparison. In the experiment, it was also observed that the self-made micro wind speed sensor had higher stability compared to the commercially available wind speed sensor (FS7.0.1L.195). The self-made micro wind speed sensor has a variation range of ±200 mm/s, while the commercially available wind speed sensor a variation range of ±1000 mm/s. The commercially available wind speed sensor (FS7.0.1L.195) can only measure the wind speed; however, the self-made integrated three-in-one microsensor can conduct real-time measurements of temperature and humidity according to the environment at that time, and use different calibration curves to know the wind speed. As a result, it is more accurate and less costly than commercially available wind speed sensors. The material cost of self-made integrated three-in-one microsensor includes chemicals, equipment usage fees, and wires. In the future, factories may install a large number of self-made integrated three-in-one microsensors in place of commercially available wind speed sensors. Through real-time wireless measurements, the self-made integrated three-in-one microsensors can achieve the control optimization of the HVAC cold air pipe’s internal environment to improve the quality of manufactured materials.

## 1. Introduction

Heating, Ventilation, and Air Conditioning (HVAC) is a system that uses mechanical equipment for ventilation. By introducing air from the outside and expelling air from the area, it could maintain the quality of indoor air and reduce moisture, odors, and pollutants in the air. Due to the need for introducing a significant amount of outdoor air, the internal environment of the HVAC cold air is affected, and wind speed, temperature, and humidity may have an internal effect. In addition, the geographical location and environment of the factory may also make a difference according to changes in temperature or short-term extreme climate (such as typhoons and heavy rain). Therefore, in order to improve the quality and reliability of manufactured materials of the factory, monitoring the internal environment of HVAC cold air ducts, such as wind speed, temperature, and humidity, and analyzing data using three essential physical quantities, are crucial research topics for finding optimal control parameters for the internal environment.

The research aims to achieve real-time wireless monitoring of important information such as wind speed, temperature, and humidity within the HVAC cold air duct, and establish a complete database. It involves planning the various types of sensors required for the internal environment of the cold air duct, their installation location and quantities, evaluating and purchasing commercially available wireless sensors of different specifications, and developing a wireless flexible three-in-one (temperature, humidity, and wind speed) microsensor for software and hardware integration. The goal is to optimize the deployment of commercially available wireless sensors and self-made wireless flexible three-in-one microsensors, and collect important information to assist in field verification. This will achieve the optimization of the internal environment control of the HVAC cold air duct and improve the quality and reliability of process materials.

Yi et al. [1] made a new type of wind speed sensor by using a flexible substrate, placed the wind speed sensor in a wind tunnel, whose resistance changed with the change of wind speed, and conducted a comparative analysis on it. Shadmand et al. [2] produced a cantilever flow sensor and tested the length, width, and thickness of the cantilever. According to the test results, the cantilever could produce more changes by reducing the thickness and increasing the length and length-to-width ratio. Sun et al. [3] studied the influence of dust and grease on the performance of wind speed sensors in harsh environments. They found that oil film with a thickness of 7–8 μm could lead to more than 40% measurement error. Son et al. [4] demonstrated that piezoelectric-based flow sensors are sensitive to changes in ambient temperature and therefore not suitable for outdoor applications. The use of capacitive-based flow sensors also results in poor sensitivity and temperature dependence due to the metal layer on the curved electrodes. Furthermore, in order to verify the effects of flow rate and temperature, a heated version was introduced, but the change in capacitance due to temperature was found to be small after experimentation. Ligeza et al. [5] placed a measuring filament in a hot wire wind speed sensor, tested the static characteristics of the wind speed sensor in a constant temperature system, and measured the relationship between gas wind speed and current. Leu et al. [6] made a heat flow sensor with micro-electro-mechanical systems (MEMS) technology, which had high resolution and high frequency responses. A metal film resistor is deposited on the surface of the sensor. Through the linear resistance coefficient of the resistance, the resistance value could be calculated through the temperature difference of the sensor, and then, the data could be obtained. Shen et al. [7] made a flexible airflow sensor using a Polyimide (PI) film. They used a varistor for sensing and placed a temperature sensor to measure resistance changes under different temperatures. Xu et al. [8] developed a heat flow sensor by combining the Internet of Things (IOT) and intelligent energy-saving buildings and applied them to HVAC. Song et al. [9] used a temperature sensor with a gold film to monitor changes in the environment temperature and used relatively sophisticated resistors and commercial amplifiers to adjust the circuit to optimize its performance. Furthermore, they corrected the sensor in the range of 27.5 °C to 32.5 °C of the thermostat to obtain the corresponding resistance values and the overall response slopes. Pan et al. [10] selected Polyaniline/graphene-polyvinyl butyral (GPANI-PVB) composite films for micro sensors. The relationship between the resistance of the cooling cycle and the thermal cycle and the temperature was tested, and the glass substrate with high temperature resistance was used to conduct the test at relatively high temperatures. It was found that the resistance would rise with the increase in temperature. Xu et al. [11] tested the temperature and relative humidity (RH) in six different combinations. They were able to obtain the temperature and RH from changes in center frequency shift and line width caused by resonance. Shinoda et al. [12] conducted a study to investigate variations in temperature measurements between nine wireless temperature sensors and two traditional wired room temperature sensors that are commercially available. They also analyzed how indoor cooling systems, load, sensor types, and locations affect these measurements. The study revealed that each sensor had varying levels of sensitivity to radiation, and the impact of radiation on the temperature readings of the wireless sensors was dependent on the difference between the black globe temperature and the air temperature. Jones et al. [13] used gold as the sensing metal and produced a temperature sensor. The bare chip was electrically heated before assembly. The hot wire was electrically heated on a Cascode Microtech Sumit 12,000 probe table and measured at different temperatures. It was found that the resistance of gold has a linear relationship with the temperature. Mehmood et al. [14] found that, under different temperatures, when a small current was applied to a Resistance Temperature Detector (RTD) and the voltage was measured at the same time, each RTD had enough time to stabilize the temperature. Li et al. [15] found that temperature changes had special effects on sensors. In practical engineering applications, the ambient temperature of the sensor may change frequently, which was different from the situation in the laboratory environment where the temperature was easily stabilized. Therefore, this may lead to measurement errors in practical applications. Pereira et al. [16] found that when the low-cost Arduino was used to collect sensor data, the correlation between its humidity measurement and reality was very high and the fitting degree of linear regression was very high. Moreover, it was believed that detectors using Arduino could be installed in a large number of buildings. Baker et al. [17] found that hysteresis was a key factor for humidity sensors, and large hysteresis was the most common problem in organic semiconductors. Guo et al. [18] conducted humidity sensing responses and voltage changes under different RH. They found that the responses and presentation of voltage value signals in each cycle were relatively uniform under several fixed RH environments. Chen et al. [19] proposed a method for correcting the error in humidity ratio measurement caused by the delay between humidity and temperature sensors. Yin et al. [20] studied dynamic water vapor adsorption at constant temperature or RH. At 20% RH, absolute humidity increased from 3.93 gm^3^ to 25.99 gm^3^ as the temperature increased from 25 °C to 60 °C, with no significant change in water absorption. Beniwal et al. [21] found that the resistance of the sensor would increase with the rise of humidity. The reason for the change of these values within a given range is that at a low relative humidity value, a physical adsorption layer of water molecules would be formed on the surface of the sensing material.

The literature measures only a single physical quantity. We developed an integrated three-in-one microsensor that can measure the three important physical quantities of wind speed, temperature, and humidity at the same time. There is a relationship between the three physical quantities. We developed an integrated three-in-one (temperature, humidity, and wind speed) microsensor using MEMS technology and use Arduino for real-time monitoring. This will enable the deployment of self-made wireless three-in-one microsensors and the collection of important information to achieve the optimization of the internal environment control of HVAC air conditioning cold air ducts.

## 2. Research Method

### 2.1. Design and Principle of Micro Wind Speed Sensors

**Figure 1:**
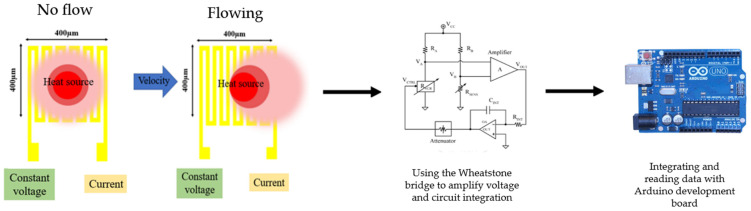
Schematic diagram of sensing principle of hot wire wind speed sensor.

At present, there are many kinds of wind speed sensors developed using MEMS technology. Generally, wind speed sensors can be divided into two categories: hot wire type wind speed sensors and calorimeter type wind speed sensors. Among them, hot wire wind speed sensors have a small size, high sensitivity, and high accuracy. Moreover, the design of its process, drive, and signal output circuit is relatively easy [22]. Therefore, hot wire wind speed sensors are suitable tools for this study. The main measuring structure of hot wire wind speed sensors is a resistance heater. The resistance heater generates a heat source as an input at a constant voltage so that the electrode becomes a resistance heater and produces a stable temperature field. When the heat of the external supply heater is fixed, the resistance value of the heater may decrease with the increase of the gas wind speed and the heat taken away, as shown in Figure 1. The length and width of the micro wind speed sensor were 400 µm × 400 µm. The minimum line width was 15 µm, the line distance was 15 µm, and the distance between the microsensors was 400 µm. It is known that gas flow can cause temperature changes and vary with the resistance, which results in a decrease in the electrode resistance when gas flows through. According to Ohm’s law (Equation (1)), when a constant voltage (V) is supplied, the lower the resistance (R), the higher the resulting output current (I). Therefore, the magnitude of the wind speed can be determined based on the proportion of the decreased voltage.

(1) $$I=\frac{V}{R}$$

### 2.2. Design and Principle of Micro Temperature Sensor

The micro temperature sensor in this study belongs to Resistance Temperature Detector (RTD). It uses gold (Au) as the electrode material and conforms to the linear equation of Equation (2) between resistivity (*ρ*) and temperature (*T*). in Equation (2), *ρ* is the resistivity at temperature *T*, *ρ*_0_ is the resistivity at reference temperature (*T*_0_), and *α* is the Temperature Coefficient of Resistance (TCR).

(2) $$\rho =\rho_{0}\left[1+\alpha \left(T-T_{0}\right)\right]$$

Equation (4) could be obtained by substituting Equation (2) into Equation (3) of resistance law, where *R*_0_ is the resistance at temperature *T*_0_, and *R* is the resistance at temperature *T*.

(3) $$R=\rho \frac{L}{A}$$

(4) $$R=R_{0}\left[1+\alpha \left(T-T_{0}\right)\right]$$

**Figure 2:**
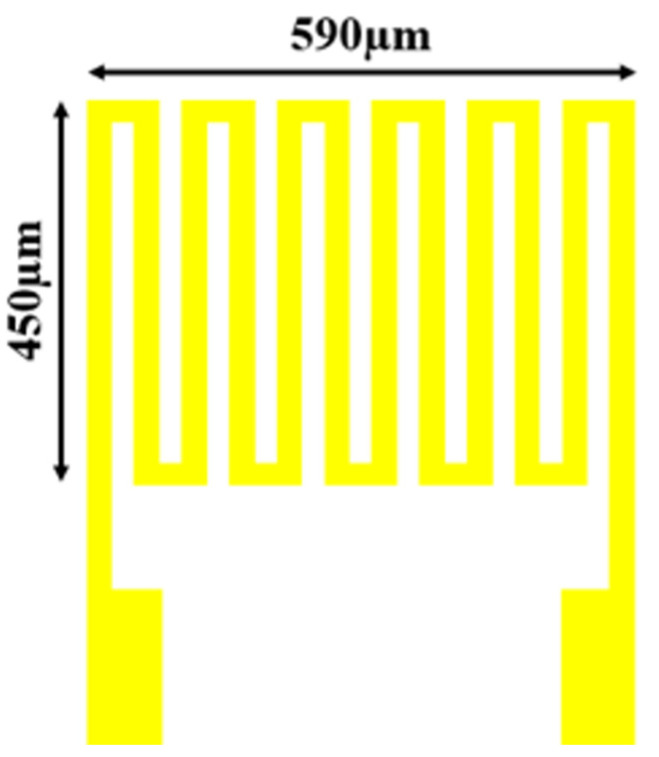
Structure design drawing of RTD.

The physical meaning of *α* here is the sensitivity of temperature (1/°C), which represents the change rate of resistance with each change of temperature per °C. It could be verified through this equation that the metal may expand and contract with the temperature change. In addition, the Steinhart-Hart Equation (SHHE) was derived by Cui et al. [23]. It further proved that the resistivity of objects may also change with temperature. In order to increase the base resistance so that changes could be measured easily, the electrode was specially designed as a snake structure, as shown in Figure 2. The length and width of the micro temperature sensor were 590 µm × 450 µm. The minimum line width was 15 µm, the line distance was 15 µm, and the distance between the microsensors was 400 µm.

### 2.3. Design and Principle of Micro Humidity Sensors

**Figure 3:**
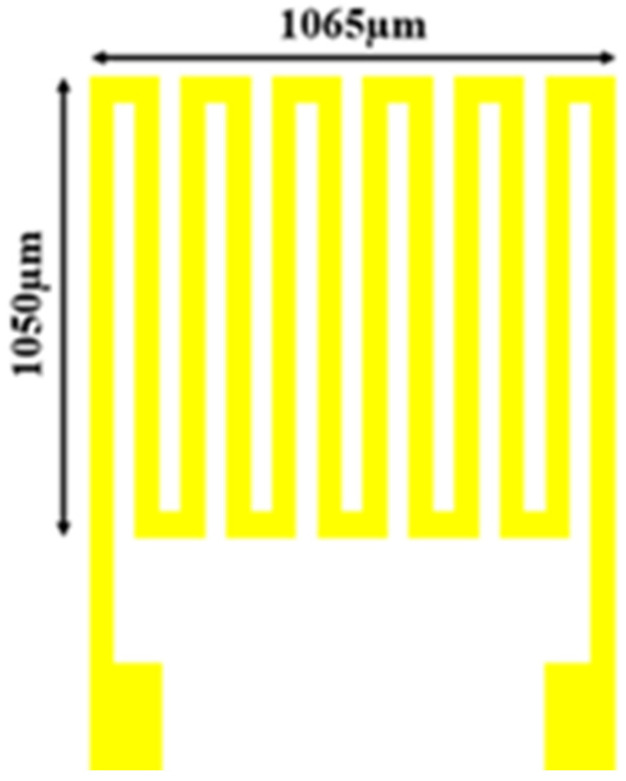
Structure design drawing of resistance humidity sensor.

The micro humidity sensor used in this study is a resistance humidity sensor. It uses a new negative photoresistance of polyimide (Fujifilm LTC^®^ PI 9305, FUJIFILM Electronic Materials Taiwan Co., Ltd., Hsin-Chu, Taiwan), which is strong in toughness, acid resistance, alkali resistance, corrosion resistance, and moisture absorption, as the moisture sensing film on the humidity sensor. Normally, such materials have non-conductive properties. When moisture absorbed by the moisture sensing film increases, the dielectric constant of the moisture sensing film may also increase with the rise in ambient humidity [24]. The increased resistance value can be directly measured through a circuit, as shown in Figure 3. The length and width of the micro humidity sensor were 1065 µm × 1050 µm. The minimum line width was 15 µm, the line distance was 15 µm, and the distance between the microsensors was 400 µm.

### 2.4. Integration of the Three-in-One Microsensor

**Figure 4:**
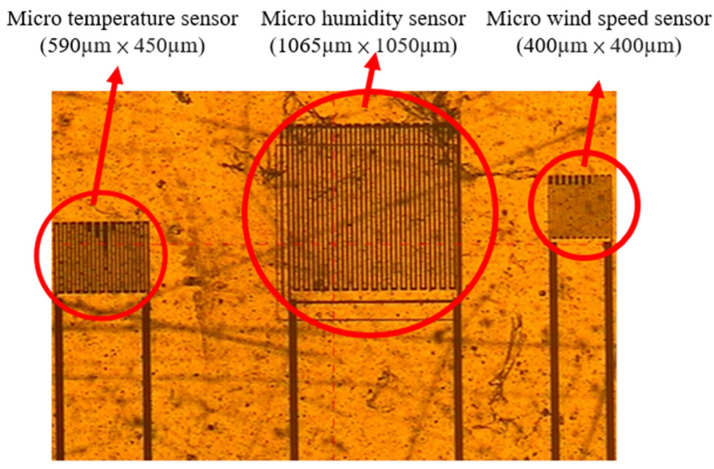
Optical microscope of the integrated three-in-one microsensor.

Figure 4 shows the optical microscope image of the integrated three-in-one microsensor. The length and width of the micro temperature sensor were 590 µm × 450 µm. The length and width of the micro humidity sensor were 1065 µm × 1050 µm. The length and width of the micro wind speed sensor were 400 µm × 400 µm. The minimum line width was 15 µm, the line distance was 15 µm, and the distance between the microsensors was 400 µm. The distance between the microsensors was very close. Therefore, it could be regarded as the temperature, humidity, and wind speed at the same point to monitor these three important physical quantities at the same time. According to the past experience of our laboratory, the sensitivity of this size is the best, and it also matches the manufacturer’s self-made circuit. The difference in position is to match the manufacturer’s mechanism design.

### 2.5. Manufacturing Process of the Integrated Three-in-One Microsensor

**Figure 5:**
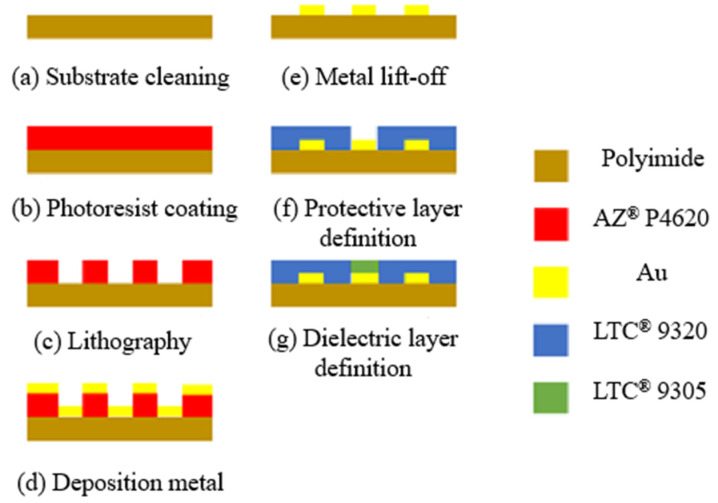
Process diagram of the integrated three-in-one microsensor.

In this study, MEMS technology was used to integrate three sensing structures, namely temperature, humidity, and wind speed. In order to make the integrated three-in-one microsensor bendable, long-term non-damage and long-term measurement in the mechanism inside the HVAC cold air pipe, this study selected anti-corrosion, high temperature resistant, stretchable, and bendable Polyimide (PI) film as the substrate of the integrated three-in-one microsensor, with the thickness of 50 µm. It uses LTC^®^ 9320 liquid polyimide (Fujifilm Durimide^®^ PI 9320, FUJIFILM Electronic Materials Taiwan Co., Ltd., Hsin-Chu, Taiwan) as the protective layer and dielectric layer. The protective layer is added to temperature and wind speed sensors to protect the circuits. An insulating layer is added for humidity sensors because the LTC 9305 is used as a humidity sensing material. If an insulating layer is added as a protective layer for humidity sensors, the humidity sensor will not be able to measure humidity. In terms of the manufacturing process, it mainly utilized deposition, lithography, and lift-off to superimpose multilayer films. Moreover, all micromechanical structures were made using thin film deposition, as shown in Figure 5. Gold (Au) has good physical and chemical properties and a relatively simple manufacturing process. Gold was used as the sensing material.

The following are the order of the processes:

(a) First, clean the PI film with organic solvents such as acetone and methanol, and then rinse it with deionized water to remove residual methanol, surface dust, and residual oil, in order to increase the adhesion of the thin film metal.
(b) Coat with AZP4620 and use photolithography to define the electrode pattern of micro temperature, humidity, and wind speed sensors.
(c) Then use an electron beam evaporator to deposit chromium and gold as adhesive layers and sensing electrode layers.
(d) After lift-off using acetone, coat with LTC 9320 as a protective layer, and then use photolithography again to define the sensing area and terminals of micro temperature and wind speed sensors, which are covered and protected by the protective layer.
(e) Coat with LTC 9305, and then use photolithography to define the sensing area of the micro humidity sensor, which is covered by a dielectric layer.

### 2.6. Assembly of the Integrated Three-in-One Microsensor

**Figure 6:**
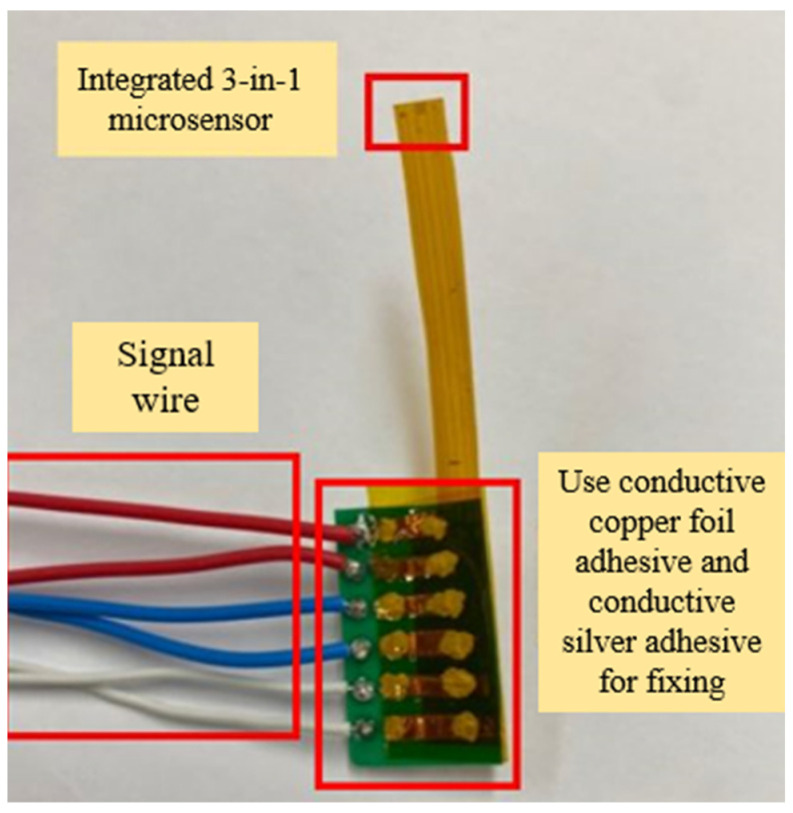
Actual photograph of signal wires and the integrated three-in-one microsensor.

The integrated three-in-one microsensor was cut to the smallest size to facilitate the placement of the internal fixing mechanism of the cold air pipe. Next, the integrated three-in-one microsensor was affixed to the ceramic substrate with conductive copper foil adhesive. Afterwards, the contact ends were strengthened and fixed with conductive silver adhesive, and the signal wires of the integrated three-in-one microsensor were welded to the ceramic substrate to connect the integrated three-in-one microsensor to the microprocessor on the computer side. The actual photo of the integrated three-in-one microsensor after signal wire welding is shown in Figure 6.

**Figure 7:**
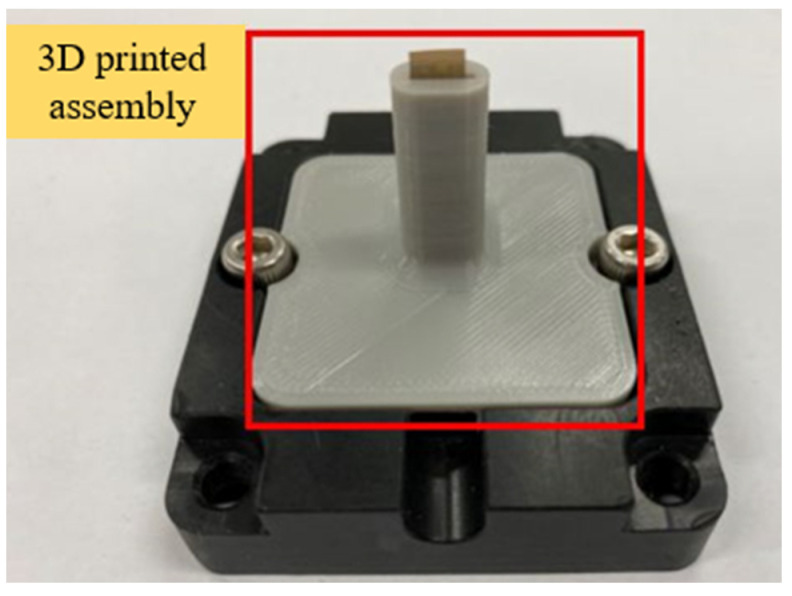
Assembly diagram of the integrated three-in-one microsensor.

The assembly of this microsensor had to conform to the fixed mechanism inside the factory’s HVAC cool air pipe and was expected to be able to resist the greasy environment inside the cold air pipe. Therefore, for the mechanism inside the cold air pipe, 3D printing was used to design the assembly as it could simultaneously fix the integrated three-in-one microsensor onto the mechanism. Finally, the integrated three-in-one microsensor was inserted into the 3D printed assembly, and the locking mechanism is shown in Figure 7. The 3D printing model is the ender 3 s1 pro from creality. The material is PETG (polyethylene terephthalate).

## 3. Results and Discussion

### 3.1. Calibration of the Integrated Three-in-One Microsensor

**Figure 8:**
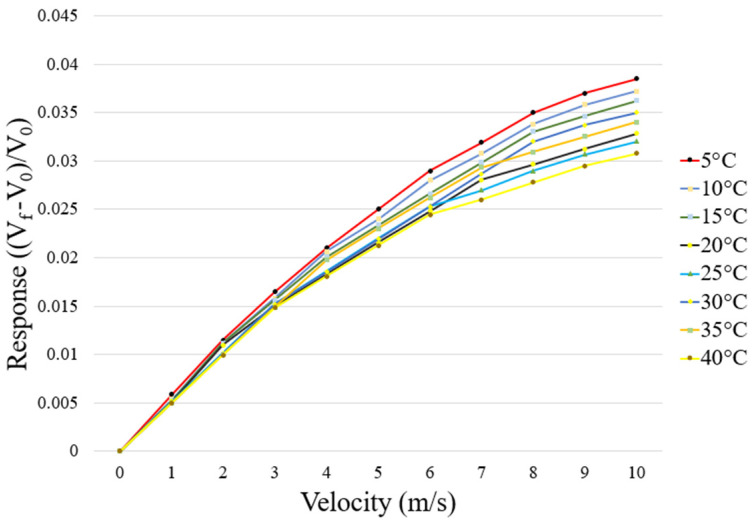
Calibration diagram of the micro wind speed sensor at different temperatures.

The wind-speed calibration range of the self-made micro wind speed sensor was 0 m/s to 10 m/s. The micro wind speed sensor might be affected by temperature. Therefore, the ambient temperature ranged from 5 °C to 40 °C during the calibration. Data were captured at wind speeds ranging from 0 m/s to 10 m/s every 5 °C and were measured repeatedly three times. In addition, through the dimensionless variation of voltage readings, the calibration diagram was obtained, as shown in Figure 8. In Figure 8, V_f_ is the voltage reading at the current wind speed, V_0_ is the voltage reading at 0 m/s, and ((V_f_ − V_0_)/V_0_) is the dimensionless variation of voltage reading.

**Figure 9:**
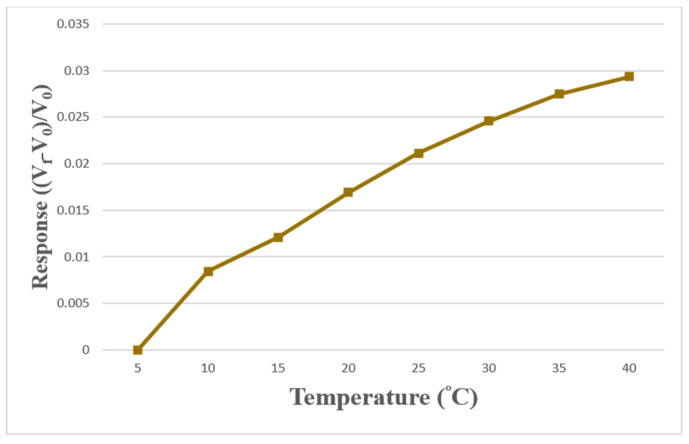
Calibration diagram of micro temperature sensors.

The temperature calibration range of the self-made micro temperature sensor was from 5 °C to 40 °C, and the interval was 5 °C. Two self-made micro temperature sensors were put into the program controllable constant temperature and humidity machine. In addition, data were collected by Arduino every 5 °C and measured repeatedly three times. The relationship curve of temperature with the original voltage value read by Arduino was obtained, and the temperature calibration curve was completed. Then, the temperature calibration curve was illustrated using the dimensionless method, as shown in Figure 9. In Figure 9, V_f_ is the voltage reading at the current temperature, V_0_ is the voltage reading at 5 °C, and ((V_f_ − V_0_)/V_0_) is the dimensionless variation of voltage reading.

**Figure 10:**
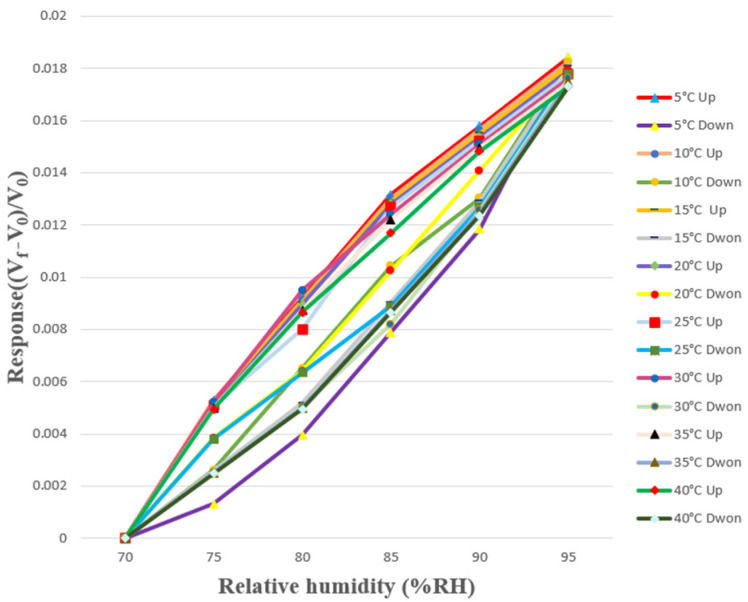
Calibration diagrams of the micro humidity sensor under different temperatures and conditions from low humidity to high humidity and from high humidity to low humidity.

The humidity calibration range of the self-made micro humidity sensor was from 70% RH to 95% RH, and the interval was 5% RH. The micro humidity sensor was put into the program controllable constant temperature and humidity machine. Since temperature might affect the resistance value of the micro humidity sensor, the calibration was carried out in the temperature range of 5 °C to 40 °C, with an interval of 5 °C. In order to ensure the accuracy of the humidity and temperature displayed by the programmable constant temperature and humidity machine, this study used a commercially available multifunctional sensor that could measure humidity and temperature at the same time for calibration. Due to the hysteresis effect of moisture-sensing materials, there might be some differences in the resistance value under the same humidity in the process of humidification and dehumidification. Therefore, in this study, the micro humidity sensor was calibrated from low humidity to high humidity and from high humidity to low humidity. Furthermore, the dimensionless variation of voltage values was used to obtain the calibration diagram, as shown in Figure 10. In Figure 10, V_f_ is the voltage reading at the current humidity, V_0_ is the voltage reading at 70% RH, and ((V_f_ − V_0_)/V_0_) is the dimensionless variation of voltage reading.

### 3.2. Comparison of Long-Time Monitoring between the Self-Made Integrated Three-in-One Microsensor and the Commercially Available Wind Speed Sensor

**Figure 11:**
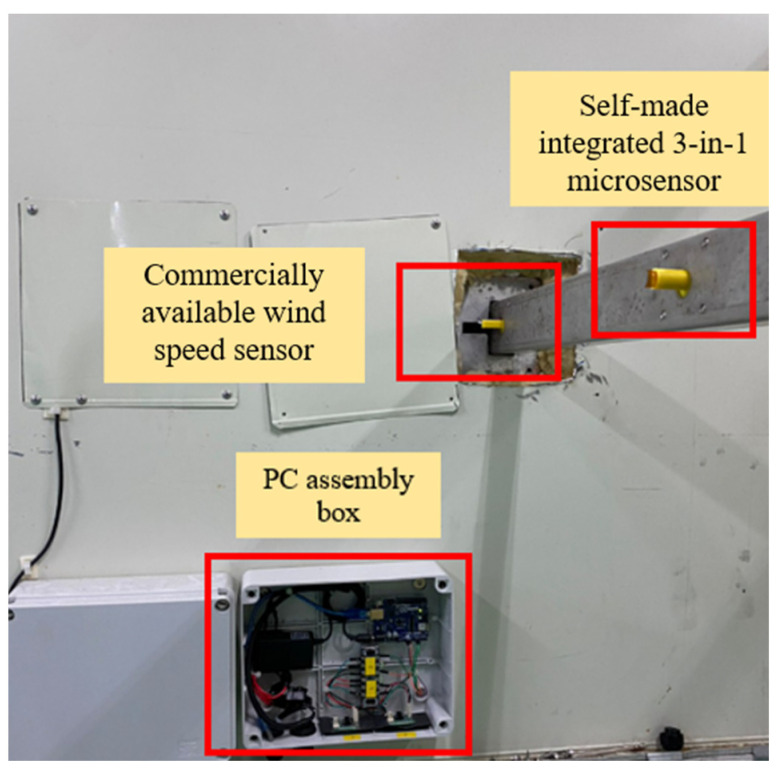
Position diagram of integrated three-in-one microsensor and commercially available wind speed sensor actually installed inside the cold air pipe of HVAC in the factory.

**Figure 12:**
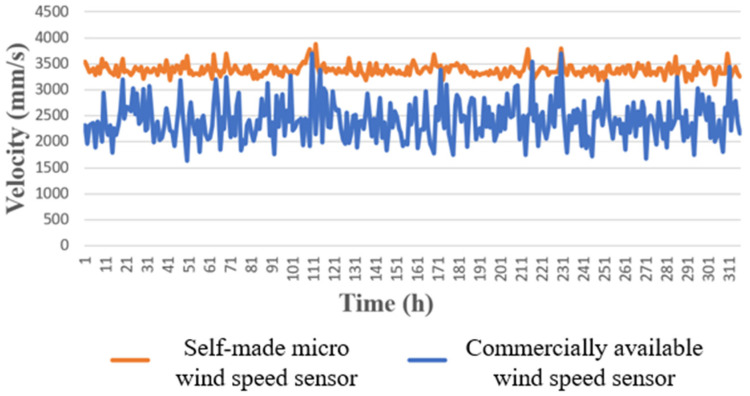
Comparison of monitoring data over 310 h between the self-made micro wind speed sensor and the commercially available wind speed sensor.

**Figure 13:**
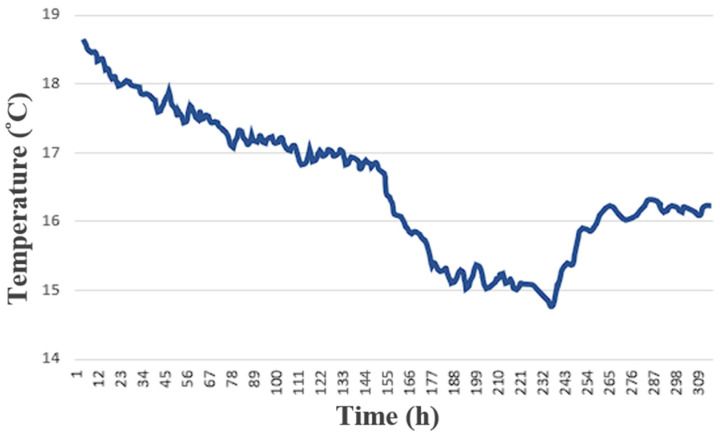
Monitoring data over 310 h of the self-made micro temperature sensor.

**Figure 14:**
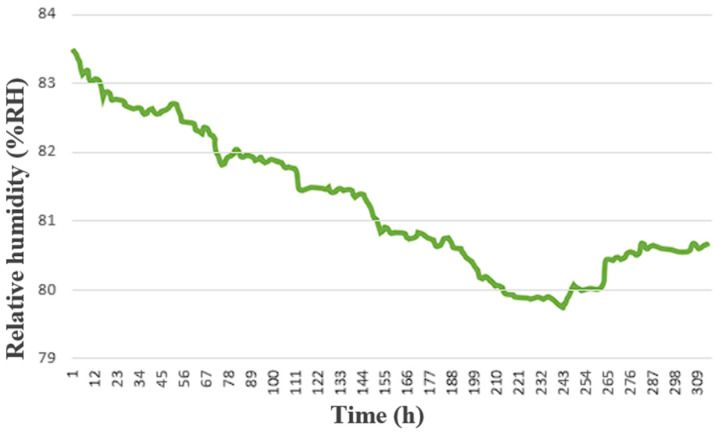
Monitoring data over 310 h of the self-made micro humidity sensor.

The self-made integrated three-in-one microsensor and the commercially available wind speed sensor were actually installed inside the HVAC cold air pipe of the factory, as shown in Figure 11. The receiving time was set to collect data every 15 s. The collected data of wind speed, temperature, and humidity were sorted and plotted, as shown in Figure 12, Figure 13 and Figure 14. The commercially available wind speed sensor (FS 7.0.1L.195) could only measure wind speeds. However, the self-made integrated three-in-one microsensor could measure the temperature and humidity in real-time according to the environment at that time and use different calibration curves to obtain the wind speed. As a result, it is more accurate than the commercially available wind speed sensor.

## 4. Conclusions

In this study, MEMS technology was used to integrate the wind speed, temperature, and humidity microsensors onto the Polyimide (PI) substrate with a thickness of 50 µm, and the polyimide (Fujifilm Durimide^®^ LTC 9320) resistant to electrochemical corrosion and acid was used as the protective layer. This integrated three-in-one microsensor has the features of a small size, high sensitivity, grease resistance, and bendability. Additionally, it offers the benefits of measuring three critical physical parameters in real-time and simultaneously, namely wind speed, temperature, and humidity. It can be placed in any position, and it is cheap and has a fast response time. By placing the self-made integrated three-in-one microsensor inside the HVAC cold air pipe of the factory together with the commercially available wind speed sensor for field monitoring and data comparison, it could be known that the self-made integrated three-in-one microsensor had higher accuracy. Moreover, the reliability and durability of the self-made integrated three-in-one microsensor were further verified over 310 h in a long-time test. The commercially available wind speed sensor (FS7.0.1L.195) can only measure wind speed, while the self-made flexible three-in-one microsensor determines wind speed based on different calibration curves for environmental temperature and humidity, making it more accurate than the commercially available wind speed sensor (FS7.0.1L.195). In the future, in terms of wireless software design, additional IC chips need to be purchased to enable an Arduino board to connect to more sensors.

## Data Availability

Not applicable.
